# The effect of a sit-stand workstation intervention on daily sitting, standing and physical activity: protocol for a 12 month workplace randomised control trial

**DOI:** 10.1186/s12889-015-1506-y

**Published:** 2015-02-15

**Authors:** Jennifer Hall, Louise Mansfield, Tess Kay, Alison K McConnell

**Affiliations:** Division of Sport, Health and Exercise Sciences, Department of Life Sciences, Brunel University London, London, Middlesex UB8 3PH England, UK; Social Sciences and Health Theme, Brunel University London, London, UK

**Keywords:** Health, Multi-component intervention, Physical activity, Process evaluation, Randomised control trial, Sedentary behaviour, Sit-stand workstation, Sitting, Standing, Workplace

## Abstract

**Background:**

A lack of physical activity and excessive sitting can contribute to poor physical health and wellbeing. The high percentage of the UK adult population in employment, and the prolonged sitting associated with desk-based office-work, make these workplaces an appropriate setting for interventions to reduce sedentary behaviour and increase physical activity. This pilot study aims to determine the effect of an office-based sit-stand workstation intervention, compared with usual desk use, on daily sitting, standing and physical activity, and to examine the factors that underlie sitting, standing and physical activity, within and outside, the workplace.

**Methods/Design:**

A randomised control trial (RCT) comparing the effects of a sit-stand workstation only and a multi-component sit-stand workstation intervention, with usual desk-based working practice (no sit-stand workstation) will be conducted with office workers across two organisations, over a 12 month period (N = 30). The multicomponent intervention will comprise organisational, environmental and individual elements. Objective data will be collected at baseline, and after 2-weeks, 3-months, 6-months and 12-months of the intervention. Objective measures of sitting, standing, and physical activity will be made concurrently (ActivPAL3™ and ActiGraph (GT3X+)). Activity diaries, ethnographic participant observation, and interviews with participants and key organisational personnel will be used to elicit understanding of the influence of organisational culture on sitting, standing and physical activity behaviour in the workplace.

**Discussion:**

This study will be the first long-term sit-stand workstation intervention study utilising an RCT design, and incorporating a comprehensive process evaluation. The study will generate an understanding of the factors that encourage and restrict successful implementation of sit-stand workstation interventions, and will help inform future occupational wellbeing policy and practice. Other strengths include the objective measurement of physical activity during both work and non-work hours.

**Trail registration:**

Clinicaltrials.gov identifier NCT02172599, 22nd June 2014.

## Background

Sedentary behaviour (taken from the Latin term *sedere*; to sit) has been operationally defined as any activity that involves an energy expenditure of ≤ 1.5 METS (metabolic equivalents), performed in seated or lying position [1]. Emerging research evidence identifies sedentary behaviour as a risk factor for negative health outcomes in the adult population; this risk is distinct and independent from risk associated with physical inactivity [2-4]. A lack of breaks in sedentary time is also related to metabolic disease risk [5,6]. For example, lipoprotein lipase is regulated differently following sedentary behaviour and physical activity; whilst a reduction in LPL activity occurs only in oxidative muscle fibres following sedentary behaviour, physical activity promotes increased LPL activity in glycolytic muscle fibres [7]. Further, a recent intervention study found that self-reported sitting time was significantly associated with a component of DNA; telomere length [8] Shortening of telomeres is associated with ageing and onset of disease [9]. This evolving evidence raises the question, “What should the person who sleeps an average of 8 hours per day [and is physically active for 30 minutes per day] do for the remaining 15.5 hours?” [10]. Thus, research and policy guidelines, such as the UK’s ‘Start Active, Stay Active’ policy are now promoting both increases in physical activity and decreases in sedentary behaviour, stating adults should “*minimise the amount of time spent being sedentary (sitting) for extended periods*” [11].

Physical activity has been defined as “any bodily movement…that results in energy expenditure” [12]. There is a well-established body of evidence supporting the health benefits of physical activity [13]. However, physical activity is a complex behaviour; participation differs among different socio-economic groups and in the contexts and environments that contribute to health inequalities [14-16]. In the UK, it is estimated that over 73% of working age adults were engaged in full time or part time employment between April 2014 and June 2014 [17] and that adults spend an average of 60% of the working day at the workplace [18]. Thus, the workplace is an ideal setting for promoting physical activity and reductions in sitting. The office is a particularly important workplace setting, given the high proportion of sedentary time in desk-based office workers. For example, an observational study measured objectively the sedentary behaviour and physical activity of 50 office-based employees for 7 consecutive days; it found that, on average, 81.8% of working hours were spent engaged in sedentary behaviours, compared to 68.9% of non-work hours. Additionally, there were fewer breaks in sedentary time, and less light-intensity activity, during working hours than during non-working hours (p < 0.001) [19]. This suggests that office-workers are more sedentary and less active during work than outside work and thus the office-based workplace is a crucial context for intervention to promote increases in physical activity and reductions in prolonged sitting. In desk-based office occupations, prolonged sitting is likely to occur in the context of travelling to work and participating in meetings, however, approximately two-thirds of workplace sitting time is spent at a desk [20].

One response to reducing sitting time in the workplace is the installation of sit-stand workstations. Sit-stand workstations offer height-adjustable desk equipment for computer screens and keyboards allowing employees a choice of desk-based working positions. Several studies have examined the contribution of sit-stand workstations to a range of sedentary behaviour, health and productivity outcomes. Previous sit-stand workstation intervention studies have seen varying reductions in the amount of time spent sitting, ranging from 66 minutes to 143 minutes per day [21]. A recent meta-analysis of activity-permissive workstations revealed that the reduction in sedentary behaviour is greater in interventions that incorporate a change to the working environment (i.e. an activity-permissive workstation), than interventions that do not [22]. Physiological research shows that using a sit-stand workstation for 185 minutes, immediately after eating, can reduce post-prandial glucose excursion by 43% [23]. A recent review of empirical studies examining the relationship between sit-stand workstations and a range of measures of productivity revealed that whilst the majority of studies show that using a sit-stand workstation had no influence on productivity, no studies showed a reduction in productivity [24]. In line with an ecological approach to behaviour change [25], initial research has shown that greater reductions in sitting time (56 min/8-hour workday) can be achieved as a result of a multi-component sit-stand workstation intervention (incorporating individual and organisational level components) compared to receiving a sit-stand workstation alone [26]. However, this needs to be replicated over a prolonged time-period (e.g. 12 months), on outcomes related to physical activity (distinct from sitting, standing and stepping). Whilst the feasibility of using sit-stand workstations has been qualitatively explored with desk-based office workers [27], outcome-focused studies dominate this research area. Understanding of the social processes that encourage and restrict standing and physical activity in the workplace is required. The absence of systematic and rigorous process evaluations of sit-stand workstation interventions will impede the wider adoption of sit-stand workstations across organisations [28,29].

### Aims

The primary aim is to determine the effect of a 12 month multi-component sit-stand workstation intervention, incorporating organisational, environmental and individual level strategies, on physical activity within the workplace (primary outcome). Secondary aims are to: determine the effect of the sit-stand workstation intervention on physical activity outside of the workplace (secondary outcome), and to determine whether the multi-component intervention is more effective than the sit-stand workstation provision alone. The process evaluation will examine the factors that influence sitting, standing and active behaviours in the workplace.

## Methods/Design

### Study design

**Figure 1:**
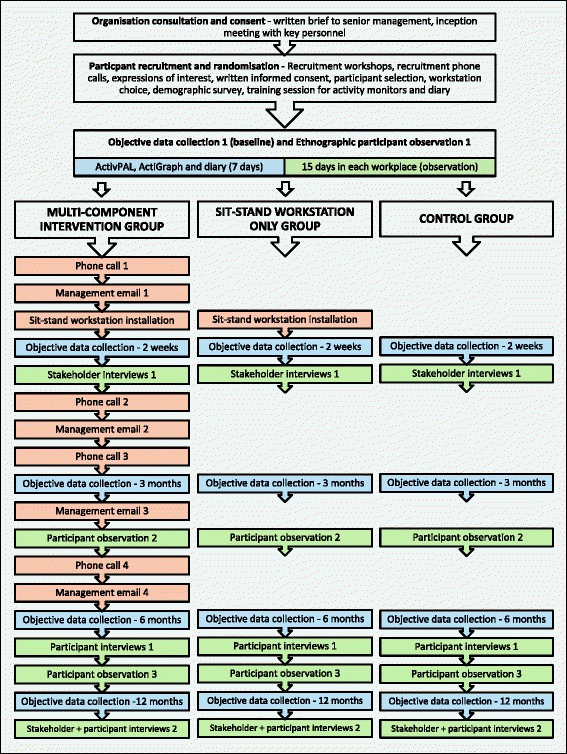
**Study overview including recruitment and study design, intervention procedures and data collection.**

An RCT comparing the effects of a sit-stand workstation only and a multi-component sit-stand workstation intervention, with usual office-based working practice (no sit-stand workstation) will be implemented. The study will be conducted with office workers across two organisations according to Cochrane recommendations [30], with ‘individuals’ being the unit of randomisation. The design employs two intervention arms: (1) a multi-component sit-stand workstation intervention (SS-MC); and (2) sit-stand workstation only (SS-O). A control arm (CG) for usual desk-based working practice (no sit-stand workstation) will also be included. Objective data collection will take place at 5 time-points over the course of 12-months: baseline (approximately one month before desk installation), and then post-installation at 2-weeks, 3-months, 6-months and 12-months. See Figure 1 for an overview of the study design and major study components. Ethics approval has been granted by Brunel University London local research ethics committee. All participants will receive a participant information sheet, which details their ethical rights, and provide written informed consent. The conduct of this study will follow the CONSORT guidelines (http://www.consort-statement.org/).

### Recruitment

#### Recruitment of organisations and study sites

Two organisations, Macmillan Cancer Support and Public Health England (PHE) will provide use of workplaces (one per organisation) and employees as participants for the study. Macmillan Cancer Support is a charitable organisation that provides support for people living with and affected by cancer. The UK Office based in Central London, UK is the worksite selected for this study. Public Health England is a governmental organisation responsible for protecting and improving the nation’s health and wellbeing, and reducing health inequalities. One PHE worksite in central London will be used in this study.

#### Recruitment of participants

Participants will be recruited via internal advertisement. At PHE, this will involve an email sent to all employees at the chosen worksite. At Macmillan Cancer Support, this involves an email sent to all employees at the chosen worksite, alongside their standard internal communication channels (Yammer page, posters). All potential participants will be required to attend a 45–60 minute recruitment workshop at their organisation. If they are unable to attend a workshop, they must engage in a 30 minute telephone conversation with the lead researcher to discuss the study requirements to be eligible to formally apply to take part in the research.

Potential participants will be invited to complete an expression of interest form that asks questions regarding age, gender, ethnicity, and disability status, as well as what interests them about sit-stand workstations, and whether they, or a close friend or family member, have ever had a cancer diagnosis*.

*Funding acquired for this study requires that some of the participants have been affected by cancer; via a personal cancer diagnosis and/or a close friend or family member being diagnosed with cancer.

#### Eligibility criteria

Participants must be full-time employees on a permanent or fixed term contract until the anticipated study end date, with no plans to leave the organisation, or be absent for an extended period (≥4 weeks). Participants must engage primarily in desk-based office-work, be present at the worksite ≥ 4 days a week, and must be at least 18 years of age. Participants must not have engaged in standing-based desk work in the four weeks prior to baseline data collection. Participants must not have been advised to avoid prolonged standing by a health professional; or be unable to stand.

Certain work teams at one of the participating organisations are excluded owing to the sensitivity of their work and current desk configuration; the current desks would need to be significantly adapted to accommodate the sit-stand equipment.

#### Participant selection

Of the potential participants that attend a recruitment workshop and complete the expression of interest form, those that do not meet the eligibility criteria will be excluded immediately. Thirty participants will then be selected strategically to take part in the research; selection will ensure the sample is representative of the range of characteristics on the expression of interest form (see above). The participants that are not selected will be put on a waiting list in case of drop-outs.

### Randomisation

Participants will be allocated randomly to one of three arms using an online randomisation programme (www.randomizer.org). Participants from the two participating organisations will be randomised separately, to achieve an equal number in each study arm in both organisations. Given the nature of the intervention, i.e. the fact that participants may or may not receive a sit-stand workstation, it will not be possible to blind participants or researchers to arm allocation. However, concealment allocation will be implemented [27].

### Trial status

At the date of manuscript submission participant recruitment has been completed, and data collection has commenced but has not been completed.

### Sit-stand multi-component intervention

#### Theoretical basis and intervention development

The intervention to be delivered to the SS-MC arm aligns with the World Health Authority’s promotion of a healthy workplace model [31]. This theoretical framework emphasises that best-practice workplace health interventions should involve an integrated approach involving organisation and individual level approaches to behaviour change [32]. This is based on an ecological definition of health and approach to behaviour change, which recognises that many factors influence health behaviour and that psychosocial influences on physical activity and sedentary behaviour vary in different contexts [25,33]. The content of the intervention will be based on previous interventions in a similar context e.g. [26,34,35] and will incorporate behaviour change techniques, including motivational interviewing [36,37]. The multi-component intervention will be refined to reflect the specific context (based on discussion with the respective organisation) and employee’s needs (following the ‘personalised inception conversation’, see below) [38]. The intervention will take effect for the first six-months following the installation of sit-stand workstations.

#### Multi-component intervention procedures

The multi-component intervention comprises organisational, environmental, and individual level elements, in accordance with the healthy workplace model [31]. For an overview of the intervention procedures within the overall context of the study please see Figure 1*.*

##### Organisational level procedures

These procedures aim to enhance the participants’ perceived level of organisational support for the sit-stand workstation intervention*.* It will consist of four emails to participants in the SS-MC arm from organisational managers who are responsible for workplace wellbeing. These emails will be sent one week before the installation of the workstation, and at weeks 6, 14 and 24 following the installation of the workstations. Emails will provide information on (a) explicit organisational support for using sit-stand workstations (motivational information) (b) the negative health impacts of sitting at work, and (c) strategies to reduce sitting and increase standing in the workplace.

##### Environmental level procedures

The environmental element will involve changes to the physical environment that are expected to facilitate reduced sitting and increased physical activity. A sit-stand workstation, i.e. height-adjustable desk equipment allowing employees a choice of desk-based working positions will be provided to participants both the SS-O and SS-MC arms for 12-months. Participants will have the choice between two models of workstation (Ergotron Workfit-A or Workfit-D, www.ergotron.com). The lead researcher will explain the differences between the two workstations at the recruitment workshop, a flyer will be given to participants, and they will have the opportunity to ‘test out’ a demo workstation within their organisation before they choose their preferred workstation. Participants will receive written and verbal instruction on the correct ergonomic posture for both sitting and standing, upon installation of the workstations.

##### Individual level procedures

Participants in the SS-MC arm will receive four 5–10 minute telephone calls (before installation of the sit-stand workstation, and at weeks 3, 10 and 19) from the lead researcher who is experienced in motivational interviewing, as a method for “enhancing intrinsic motivation to change, by exploring and resolving ambivalence” [39]. These telephone calls will engage participants in conversations related to the sit-stand workstations and physical activity that will follow the principles of engaging, guiding and evoking to motivate and support participants to reduce their inactivity and increase activity, primarily at the workplace.

### Data collection

The research design is underpinned by the standard evaluation framework for physical activity programmes, incorporating both outcome and process evaluation data collection [40]. See Figure 1 for an overview of the data collection procedures.

#### Participant profile data

Profile data will be collected, through the expression of interest form (detailed earlier) and a short survey undertaken at a monitoring training session, before the installation of the sit-stand workstations. Questions on the short survey will include participants’ height, weight, job title, household composition, personal monthly income, level of education, and sexuality.

#### Outcome data

Primary outcomes are: daily time spent sitting, standing, in light and moderate-vigorous physical activity, the average number of sit-stand transitions per hour, number of prolonged sitting bouts ≥30 min, and total physical activity energy expenditure during work hours. Secondary outcomes will be the same, but over the course of the entire day (see Table 1)*.* These will be measured at baseline (approximately one month before sit-stand workstation installation) and four subsequent time-points following workstation installation; two-weeks, 3-months, 6-months and 12-months. Each data collection period will be 7-days. Sitting, standing and sit-stand transitions will be assessed using an ActivPAL3™ micro monitor (PAL Technologies Limited, Glasgow, UK) and light, moderate and vigorous physical activity will be assessed using an accelerometer (ActiGraph GT3X+, ActiGraph, Pensacola, FL, USA). The ActivPAL3™ provides accurate measures of sitting time and sit-to-stand transitions per hour and sit-stand transitions, when compared to direct observation in office-based free-living environments [41,42]. The ActivPAL3™ will be worn continuously on the centre of the right thigh, following insertion into a nitrile sleeve and wrapping in a waterproof dressing. Participants will be given extra materials to change the dressing when required. Participants will be requested to wear the monitor continuously for each 7-day data collection period. The ActiGraph GT3X+ is a tri-axial accelerometer that accurately classifies physical activity intensity in free-living environments [43]. It will be worn on the hip via an elastic belt during waking hours only, excluding time spent engaged in water-based activities. Participants will be asked to record non-wear time of both monitors, and time spent at the workplace for all 7-day data collection periods.

**Table 1:** **Study outcome measures taken at baseline, 2 weeks, 3 months, 6 months and 12 months**

	***Working hours***	***Total hours***
**ActivPAL3** **™**		
• Sitting time	✓	✓
• Number of prolonged (≥30 min) sitting bouts	✓	✓
• Number of sit-to-stand transitions	✓	✓
• Standing time	✓	✓
**ActiGraph (GT3X+)**		
• Time in light physical activity (min)	✓	✓
• Time in moderate physical activity (min)	✓	✓
• Time in vigorous physical activity (min)	✓	✓
• Time in MVPA in 10-min bouts (min)	✓	✓
• Counts per minute	✓	✓

#### Process evaluation

In alignment with the Medical Research Council guidelines on evaluating complex interventions [38], the RCT is accompanied by a systematic and rigorous process evaluation to gain an understanding of how sit-stand workstation interventions work in practice [44]. Process evaluations are particularly important in multi-site interventions to explore differences in implementation and outcomes [45].

##### Activity diaries

Activity diaries will be used to determine the type and context of active and inactive behaviours, to facilitate understanding of the factors that influence sitting, standing and physical activity in the workplace context. Participants will be asked to record the behaviour that they spent the most time doing during each hour, during each 7-day objective data collection period. A text message, reminding participants to complete their activity diary, will be sent to participants once daily throughout each data collection period. Text message models have shown success in increasing compliance to data collection [46].

##### Ethnographic participant observation

This method will be used to understand organisational culture, placing importance on the perceptions of the people under study within their usual environment [47]. The ethnographic participant observation will provide insight into the context within which the intervention is taking place; this will elicit understanding of the feasibility and acceptability of sit-stand workstation interventions, and the social processes that underpin use of the sit-stand workstations and physical activity in the workplace. The participant observation involves the lead researcher taking a volunteering role within the participating organisations. There will be three observation phases within each worksite; before the installation of sit-stand workstations, 4–5 months following the installation of sit-stand workstations, and 10–11 months following the installation of sit-stand workstations. The lead researcher will spend approximately 12 full working days at the workplace during each phase, engaging in work tasks related to physical activity and/or workplace health, set by the volunteering organisation.

##### Key stakeholder and participant interviews

Qualitative interviews will enable explorations of perceptions and meaning to increase understanding of a particular phenomenon [48]. Thus, interviews will be used within this study to delineate the perceptions of both key stakeholders and participants in relation to sit-stand working and physical activity in the workplace, to understand the factors that influence the success (or otherwise) of the sit-stand workstation intervention in influencing sitting and physical activity. Specifically, interviews with participants will explore processes that influence use of sit-stand workstations, such as experiences of standing, relationships with other employees and working identities, and will last approximately 60 minutes. These will take place with 12 participants at two time points; 7-months and 12-months following the installation of sit-stand workstations. Interviews with key stakeholders and decision makers in the implementation process from the participating organisations, will elicit understanding of the feasibility, acceptability and sustainability of the sit-stand workstation intervention. Interviewees will include staff working in employee wellbeing, human resources, IT, health and safety, and facilities, as well as estates personnel and senior directors. Approximately ten stakeholders from each organisation will engage in 45 minute telephone interviews, within two months of sit-stand workstations being installed.

### Sample size

A formal power analysis to determine sample size was not conducted as the threshold for meaningful reductions in sitting is unknown. The proposed sample size of the present study (N = 30) is similar to previous pilot studies utilising sit-stand workstations in office environments that have reported significant changes in outcomes related to sitting, standing and stepping e.g. [35,49,50].

### Data analysis

#### ActivPAL3™

The monitor records the exact duration of each bout of sitting (or lying), standing, and stepping. Data will be downloaded and processed further using ActivPAL3™ proprietary software, PAL Analysis 7.

#### ActiGraph GT3X+

Activity counts will be recorded at 1-second intervals. Data will be downloaded and processed using proprietary software, ActiLife 6. Time spent in light, moderate and vigorous activity will be calculated by classifying vector magnitude counts according to the cut-points developed by Troiano; light activity is considered 101 to 2019 counts per min, moderate activity is considered 2020 – 5998 counts per min, and vigorous activity ≥5999 counts per min. These cut-points have been validated in the general adult population [51].

#### Non-wear time criteria

ActiGraph GT3X+ non-wear criteria will be developed based on acceptable standards and examination of the data, as different criteria have been shown to impact the final sample size and outcome variables [52]. By comparing the ActiGraph GT3X+ to the ActivPAL3™ output, it will be possible to ascertain which criteria are most accurate for the data set.

#### Qualitative data analysis

Brief ethnographic field notes will be recorded throughout the day during the participant observation phases, with expanded field notes being written at the end of each day of participant observation. All interviews will be recorded using an Olympus LS-11 Dictaphone and transcribed ad verbatim. Qualitative data will be analysed using thematic content analysis via NVivo 10 software. Thematic analysis reports detail-rich data by identifying, analysing and interpreting patterns within the data [53]. Analysis will initially involve reading and re-reading the field notes and transcripts to become fully immersed in “the details and specifics of the data” to allow unearthing of patterns, themes and interrelationships ([54] p.362). Data will then be coded before searching for, identifying, reviewing and defining themes [55].

### Statistical methods

Two-way repeated measures ANCOVA will be conducted using IBM SPSS Statistics 20 to compare the outcome measures between study arms and across the intervention (between data collection time points). Relevant covariates will be controlled for, including body mass index.

## Discussion

Given the accumulating evidence of the health risks of prolonged sitting [2-8], and the epidemiological evidence that illustrates the high occurrence of prolonged sitting in office-based workplaces [18-20], high quality intervention studies are necessary to provide an evidence-base of ‘what works’ to reduce prolonged sitting and increase activity in the workplace. It is not only necessary to understand *whether* an intervention is effective, but also to understand *why* an intervention is, or is not, feasible, effective or sustainable. Accordingly, the contiguous systematic and rigorous process evaluation integral to this study represents an innovation within this area of research. Eliciting understanding of the social processes that underpin use of sit-stand workstations and physical activity within the workplace is crucial for understanding how to influence behaviour. A strength of the present study is that it will create a comprehensive picture of total physical activity using two objective measures; these will capture sitting, standing, and light, moderate and vigorous physical activity, alongside an activity diary, that will provide contextual information. The latter will include the type and purpose of the activities and the inactive behaviours undertaken. An understanding of the influence of the sit-stand workstation intervention on physical activity during non-work hours will also be permissible, as physical activity will be measured over the course of the entire day. A further strength is the long-term nature of the evaluation, which permits assessment of the sustainability of sit-stand workstation approaches for reducing prolonged sitting and increasing physical activity in the workplace [35]. The findings of this pilot study will provide evidence to guide future research, as well as the development of guidelines and policy to optimise the promotion of workplace health and wellbeing.

## Abbreviations

CG: Control group
PHE: Public Health England
RCT: Randomised control trail
SS-MC: Multi-component sit-stand workstation intervention
SS-WO: Sit-stand workstation only
